# Outlier Loci Detect Intraspecific Biodiversity amongst Spring and Autumn Spawning Herring across Local Scales

**DOI:** 10.1371/journal.pone.0148499

**Published:** 2016-04-06

**Authors:** Dorte Bekkevold, Riho Gross, Timo Arula, Sarah J. Helyar, Henn Ojaveer

**Affiliations:** 1 Technical University of Denmark, National Institute of Aquatic Resources, Charlottenlund, Denmark; 2 Estonian University of Life Sciences, Institute of Veterinary Medicine and Animal Sciences, Department of Aquaculture, Tartu, Estonia; 3 University of Tartu, Estonian Marine Institute, Pärnu, Estonia; 4 Institute for Global Food Security, Queen’s University Belfast, Belfast, United Kingdom; University of Iceland, ICELAND

## Abstract

Herring, *Clupea harengus*, is one of the ecologically and commercially most important species in European northern seas, where two distinct ecotypes have been described based on spawning time; spring and autumn. To date, it is unknown if these spring and autumn spawning herring constitute genetically distinct units. We assessed levels of genetic divergence between spring and autumn spawning herring in the Baltic Sea using two types of DNA markers, microsatellites and Single Nucleotide Polymorphisms, and compared the results with data for autumn spawning North Sea herring. Temporally replicated analyses reveal clear genetic differences between ecotypes and hence support reproductive isolation. Loci showing non-neutral behaviour, so-called outlier loci, show convergence between autumn spawning herring from demographically disjoint populations, potentially reflecting selective processes associated with autumn spawning ecotypes. The abundance and exploitation of the two ecotypes have varied strongly over space and time in the Baltic Sea, where autumn spawners have faced strong depression for decades. The results therefore have practical implications by highlighting the need for specific management of these co-occurring ecotypes to meet requirements for sustainable exploitation and ensure optimal livelihood for coastal communities.

## Introduction

Intra-specific variation is an important component of biodiversity and it is a central issue to determine evolutionary divergent population units in conservation and management. For instance, ecosystem and hereunder fisheries management hinges on the ability to estimate population- (or ‘stock’-) specific dynamics and rates of exchange of individuals among local demes. Population genetic tools have been successfully applied to describe and monitor population structure across a range of marine taxa [1]. However, many marine species display traits such as large effective population size, high fecundity and high levels of dispersal and gene flow that impede the detection of local demographics using standard population genetic approaches (e.g. [1,2]). An alternative approach is to use variation in genetic markers which reflect adaptation to ecologically divergent local habitats [3]. These markers exhibit elevated levels of genetic divergence, and can be used as a ‘tag’ for identifying reproductively isolated populations when neutral marker variation is uninformative (e.g. [4]). The detection of these ‘outlier loci’ linked to selective processes has been facilitated by relatively easy access to genome-wide sequence data, and extends to non-model species.

Atlantic herring, *Clupea harengus*, is a classic example of a widely distributed marine fish exhibiting very large and fluctuating population sizes [5] with weak genetic structuring across the North Atlantic [6–11]. Although genetic structure is evident, most inference on population divergence stems from analysis of genetic markers that are ‘outlier loci’, i.e. exhibiting variation that is not statistically reconcilable with expectations under a neutral model (e.g. [12–15]), and thus may reflect local selective responses. Thus, a suite of recent genomic sequencing studies indicate the existence of substantial genetic divergence at these loci even among individuals sampled across geographically limited (<300 km) scales ([14, 16–17], but also see [18]).

The Baltic Sea is a semi-enclosed, brackish, oceanographically highly heterogeneous and dynamic environment. Due to its young geological age and spatially contrasting environmental conditions adaptive evolution has been fast, and several species live at the limits of their physiological tolerance (e.g. [19, 20]) exhibiting relatively low genetic diversity [20]. Neutral marker based studies of herring report weak population structure in the Baltic Sea [6,10,12,14,21]. Nonetheless, within this environmentally highly heterogeneous area several demographically disjoint spring and autumn spawning herring sub-populations have been described, based on differences in life-history traits, otolith appearance, morphology and growth ([22] and references therein). Most Baltic herring mature at two years of age and spawn inshore in spring (April-June). In contrast, Baltic autumn spawning herring mature at 3–4 years and spawn offshore in August-November, at relatively deeper and broader depth ranges [23]. While metamorphosis of the spring spawning herring larvae takes place in the same year as spawning, the larvae of autumn spawning herring mostly overwinter at a larval stage and metamorphose the following spring [23].Thus, autumn and spring herring exhibit major differences in several key traits and may be characterised as ecotypes. However, based on a suite of traits including otolith growth patterns and genetic markers, spawning time appears to be a plastic trait in herring [24–25] and individuals may even shift spawning time between years [26]. Spawning time may therefore not be a population delineating factor *per se* [27] and the demographic connectivity and ultimately evolutionary linkages between Baltic herring spawning at different times remain un-described. An example of co-occurring spring- and autumn spawning ecotypes is Gulf of Riga herring. The Gulf of Riga is a relatively autonomous sub-system in the Baltic Sea. Herring is economically the most important species by far in this region, and has been systematically investigated since the late 1940s [28].

In the present study we used both neutral and ‘selective outlier’ marker information generated for temporally replicated samples from three spawning locations within the Gulf of Riga in conjunction with previously published data to test the hypotheses that autumn and spring spawning herring in the gulf constitute a single genetic population and whether ecotypes co-vary with outliers at broader geographic scales. We compared inference from neutral markers that allow insights into demographic parameters following neutral model expectations, with outlier markers to assess levels of ecotype differentiation and demographic connectivity. We show that outlier markers, but not neutral markers, support a link between ecotype and genotype. Also, even disjoint autumn spawning populations display convergent allele frequencies at specific loci. Our results contribute both towards a general understanding of genetic processes in marine fish populations, and specifically point to the importance of incorporating ecotype variation in fisheries management.

## Material and Methods

### Samples

**Fig 1:**
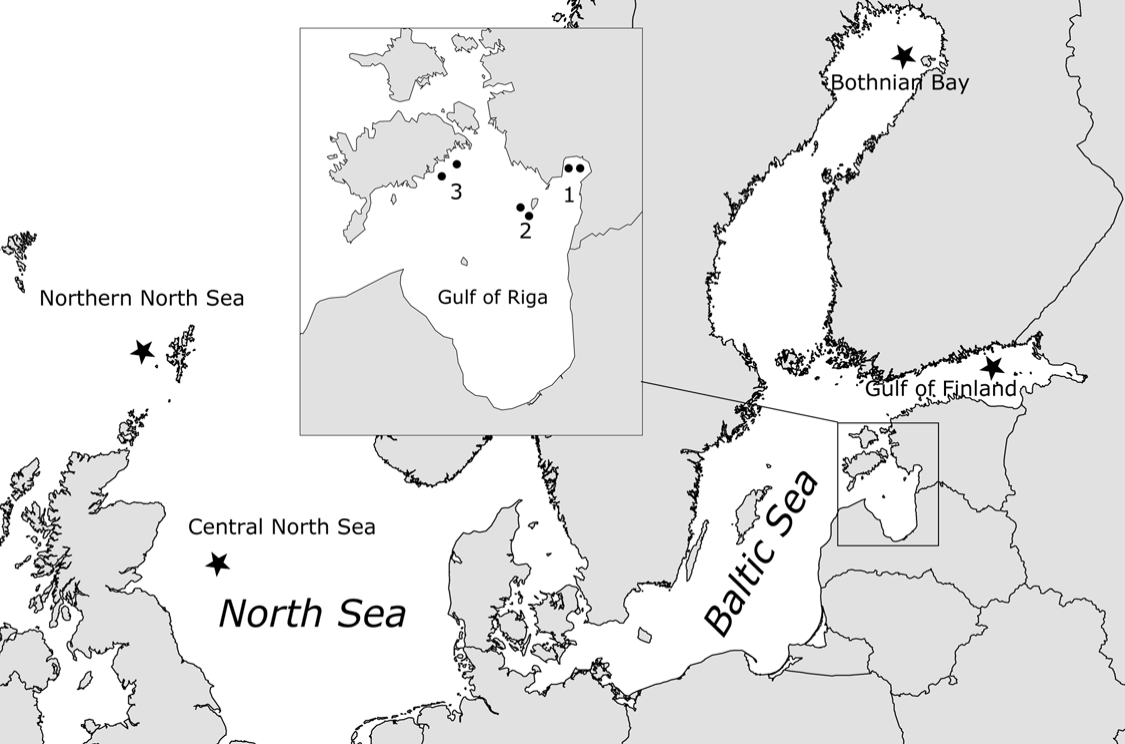
*Clupea harengus* sampling locations in the Gulf of Riga (inset) and adjacent areas in the Baltic Sea and the North Sea. Numbers show sampling locations for respectively 1. Pärnu Bay, 2. Kihnu Island, and 3. Saaremaa. Four stars show origin of collections from [14].

Spring spawning herring (here abbreviated SS) were collected from commercial trapnet catches in Pärnu Bay in the Gulf of Riga (abbreviated GoR): Samples were expected to represent mostly local GoR SS but may have included SS transient migrants from elsewhere. Autumn spawning herring (abbreviated AS) were obtained from gillnets operating in two spawning areas: near Kihnu Island, and at the southern coast of the Island Saaremaa (Fig 1). The AS feeding in the northeastern Baltic Proper spawn around the coast of Saaremaa, whereas the GoR AS utilize spawning areas around Kihnu Island [29]. All samples represented dead fish obtained from commercial fishermen and no specific permissions were required for obtaining them. None of the material was from endangered or protected species. All collected fish (Table 1) were measured for total length and total weight, aged from otoliths and their sex and maturation stage was determined using methods developed for herring [30]. The collected samples contained both fully ripe herring as well as herring with developing gonads. The latter were presumably non-spawning fish, representing either ‘skipped spawners’ [31] of local origin or migrants not belonging to local spawning components. A sample of fin tissue was collected for each fish and stored in 96% ethanol until molecular analysis.

**Table 1:** *C*. *harengus* samples in the analysis. Numbers of genotyped fish; numbers in brackets show numbers of fish per sample in pre-spawning stage (i.e. non-spawning).

Location	Sample size (# non-spawning)	Collection date	Spawning type	Markers typed (M = micro, S = SNP)	Latitude/longitude
Pärnu Bay	48 (4)	24.04.2014	Spring	S,M	58° 17,195’ N/24° 21,967’ E
	48 (1)	30.04.2014	Spring	S,M	58° 16,139’ N/24° 26,337’ E
Kihnu	48 (1)	11.09.2014	Autumn	S,M	58° 02,253’ N/23° 50,320’ E
	48 (9)	01.10.2014	Autumn	S,M	58° 01,697’ N/23° 50,199’ E
Saaremaa	48 (0)	03.09.2014	Autumn	S,M	58° 02,253’ N/23° 50,320’ E
	48 (2)	18.09.2014	Autumn	S,M	58° 02,253’ N/23° 50,320’ E
Bothnian Bay§	30 (NA)	Jun-2009	Spring/summer	S	65° 02’37.75 N/24° 45’13.17 E
Gulf of Finland§	23 (NA)	May-2009	Spring	S	60° 10’01.54 N/25° 37’14.67 E
C North Sea, Banks§	30 (NA)	Aug-2009	Autumn	S	56° 29’31.16 N/0° 33’32.15 E
N North Sea, Shetland§	30 (NA)	Aug-2009	Autumn	S	60° 04’34.07 N/1° 25’17.88 W

§ Data from [14].

### Molecular analyses

DNA was extracted from all 288 fish using E.Z.N.A. Tissue DNA kit (Omega Bio-Tek, Norcross, GA, USA). A NanoDrop Spectrophotometer (Thermo Fisher Scientific Inc.) was used to ensure adequate quality and quantity of DNA prior to genotyping.

All individuals were genotyped for Single Nucleotide Polymorphism (SNP) and microsatellite marker variation as follows. Variation was screened for 96 SNP loci selected from [32] to maximise clustering and genotyping success, and exhibiting minor allele frequencies > 0.02 in previously genotyped Baltic Sea samples. Based on a set of 281 SNPs including those analysed here, Limborg et al. [14] reported population structure across NE Atlantic populations, including six Baltic spring spawning populations. They found that sixteen loci exhibited evidence of being ‘selective outliers’, that is, loci exhibiting population divergence above neutral expectations, suggesting that they are either located in or are in linkage disequilibrium with genes under divergent selection [33]. Although such loci may not always behave as outliers we hypothesised that they would exhibit non-neutral behaviour in collections tested here. Fourteen of these outlier loci were included in our analysis. Two were excluded as they exhibited minor allele frequencies <0.02 in previously examined Baltic Sea populations [14] and their information content therefore was expected to be low. Individuals were additionally screened for 19 transcriptome-derived di-, tri-, and tetra-nucleotide microsatellite loci selected from [34]. These loci were expected to reflect neutral demographic processes as none exhibited evidence of ‘selective outlier’ behaviour in spring or summer spawning populations across the Baltic Sea [21]. Locus names are listed in S1 Table. To compare local (GoR) with Baltic Sea scale genetic structure, as well as with extant AS populations, we compared our SNP marker data with data from [14] for the same set of loci typed in two additional Baltic SS populations, and in two AS populations from the western North Sea (Fig 1).

For SNPs, PCR amplification and genotyping were performed in 96.96 Dynamic Arrays using the Fluidigm IFC thermal cycler and BioMark instruments with SNPtypeTM chemistry. Genotypes were scored using the BioMark Genotyping Analysis software (Fluidigm, San Francisco, California, USA). SNP genotypes obtained using a different genotyping platform from [14] were validated across platforms, as described in [15]. Microsatellite loci were amplified in two multiplex (11-plex and 8-plex) PCR reactions. The 10 μl PCR reaction consisted of ca. 20 ng template DNA, 1x Type-it Multiplex PCR Master Mix (QIAGEN, Germany), and 150 to 200 nM of each primer. Forward primers were labelled fluorescently by 6-FAM, ATTO 550, ATTO 565 or Yakima Yellow. Amplifications were carried out in a Biometra Professional Thermal cycler with an initial heat-activation at 95°C for 5 min followed by 28 cycles of denaturation at 95°C for 30 s, annealing at 56°C for 90 s, extension at 72°C for 30 s, and a final extension for 30 min at 60°C. Multiplex PCR products were electrophoresed on an Applied Biosystems 3500 Genetic Analyser (Life Technologies, USA) and the loci were genotyped using GeneMapper v.5 software (Life Technologies, USA).

### Statistical analyses of genetic variation

Conformance with Hardy-Weinberg proportions (HWE) and gametic phase equilibrium (LD) were examined for all markers and collections using Genepop [35]. Observed and expected heterozygosity and allelic richness were estimated per locus and sample using the R-package *hierfstat* [36]. Differentiation overall and between pairs of samples was estimated using Ɵ [37], and statistical significance of population differentiation was examined using exact G-tests in Genepop. Table-wide statistical significance levels were adjusted using False Discovery Rate, FDR, following [38]. To assess evidence for consistency in which markers behaved as outliers in the samples collected here with those in [14], we performed an outlier detection analysis, applying the Bayesian likelihood method implemented in the software BayeScan (http://www.cmpg.unibe.ch/software/bayescan/), adopting the same settings as [14]. A log10 Bayes factor above 0.5 was considered evidence for outlier behaviour [39].

For exploration of the genetic structure reflected in the SNP data within and among SS and AS collections from the Baltic and the North Sea, we applied discriminant analysis of principal components, DAPC [40], implemented in R-package *adegenet* [41]. We first used the find.clusters() function to run K-means clustering of the individual genotypes for K = 1–40. The best supported number of clusters was estimated through comparison of the Bayesian Information Criterion (BIC) for the different values of K. We then described the relationships between the inferred clusters using the dapc() function. This function constructs synthetic variables, discriminant functions (DFs) that maximise variation between, while minimising variation within, groups and computes coordinates along these functions for each individual. We retained the first hundred principle components (PCs) from the preliminary data transformation step, as this was indicated to be the optimal number based on the optim.a.score() function. From the derived DFs, we obtained posterior cluster membership probabilities for each individual to the K clusters, and estimated the contributions of individual loci to each of the PCs of the analysis. The analysis was first performed using information for all SNP markers, and was then repeated for information for non-outlier SNPs, thus using neutral marker information to assess demographic relationships among populations.

Finally, to assess potential bias of including non-spawning individuals, we used *adegenet* to perform a clustering analysis where these fish were excluded from the initial clustering analysis and then tested the clustering (‘assignment’) of these hold-out fish against a model fitted to spawning fish only.

## Results

### Estimates of genetic variation

A total of 287 and 279 individuals were genotyped successfully for microsatellite and SNP loci, respectively. For microsatellites, the total numbers of alleles per locus varied between four and 24 (S2 Table), and global mean allelic richness was 7.12. One microsatellite locus, *Her114*, exhibited heterozygote deficiency and statistically significant deviation from HWE proportions in three collections (following correction for multiple testing). This locus was therefore excluded from further analysis. For SNPs, one locus (*Cha_8386*.*6_423*) suffered technical error (lack of clustering) in genotyping and was excluded from further analysis. Generally, levels of H_e_, H_o_ and allelic richness were similar across all collections (S2 Table). Three of the 678 (0.044%) tests for HWE were statistically significant following FDR correction indicating low risk of biased allele frequency estimates. Nine locus comparisons out of 6328 (0.001%) exhibited statistically significant deviation from gametic phase equilibrium following correction for FDR. As no deviation occurred across multiple samples LD was not expected to bias results.

**Fig 2:**
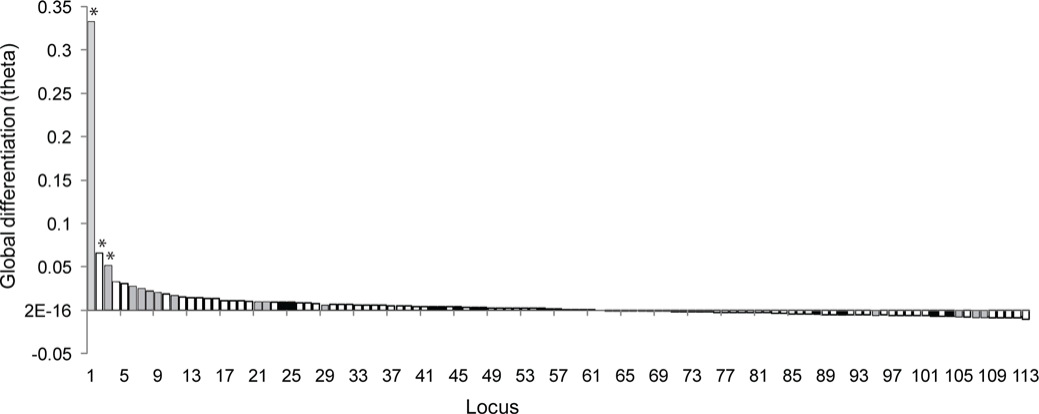
Locus specific differentiation across six GoR samples. Global differentiation (Weir-Cockerham’s ϴ) for 18 microsatellite (dark grey bars) and 95 SNP (open bars) loci ranked by ϴ. Hatched bars indicate SNP loci identified as selected outliers in [14] and asterisks above bars indicate three loci identified as outliers in this study. See S1 Table for locus ID. Global ϴ across loci and samples was 0.0066.

Combining information for all loci, pairwise sample divergence ranged between 0.000 and 0.013 among GoR samples. Statistically significant differentiation was observed for several pairs of collections; all involving comparisons between SS and AS (Table 2). For individual loci, four SNPs (Cha_15360.2_279, Cha_381.2_437, Cha_16330.7_357, Cha_2884.1_367) and one microsatellite locus (Her142) exhibited statistically significant differentiation between SS and AS collections following correction for multiple testing. One SNP locus, Cha_7833.1_97, exhibited low but statistically significant differentiation between AS from Kihnu and Saaremaa, hinting at differentiation between gulf- versus open sea components. Generally, SNPs exhibited higher global differentiation than microsatellites, and save a few exceptions, previously identified outlier SNPs generally exhibited higher than average differentiation within the GoR (Fig 2). The outlier analysis detected genetic differentiation to be above expectations for three SNP loci, two of which were also outliers in [14] (Fig 2). When microsatellite data were analysed alone, global population differentiation was estimated at 0.0005, varying between 0 and 0.005 across pairwise comparisons of collections, none of which were statistically significant across loci.

**Table 2:** Pairwise differentiation (ϴ, above diagonal) and P-values for Fisher’s tests for differentiation (below diagonal) for SNP and microsatellite markers combined. Statistically significant divergence following FDR correction is shown in bold. SS and AS indicate spawning in respectively spring and autumn.

	Pärnu Bay 1	Pärnu Bay 2	Kihnu 1	Kihnu 2	Saaremaa 1	Saaremaa 2
Pärnu Bay 1 24.04.2014 (SS)		0.0022	**0.0081**	**0.0120**	**0.0096**	**0.0105**
Pärnu Bay 2 30.04.2014 (SS)	0.2342		**0.0057**	**0.0128**	**0.0124**	**0.0106**
Kihnu 1 11.09.2014 (AS)	<0.0001	<0.0001		0.0014	0.0027	0.0000
Kihnu 2 01.10.2014 (AS)	<0.0001	<0.0001	0.5462		0.0053	0.0016
Saaremaa 1 03.09.2014 (AS)	<0.0001	<0.0001	0.3599	0.3544		0.0031
Saaremaa 2 18.09.2014 (AS)	<0.0001	<0.0001	0.0519	0.7336	0.2072	

**Fig 3:**
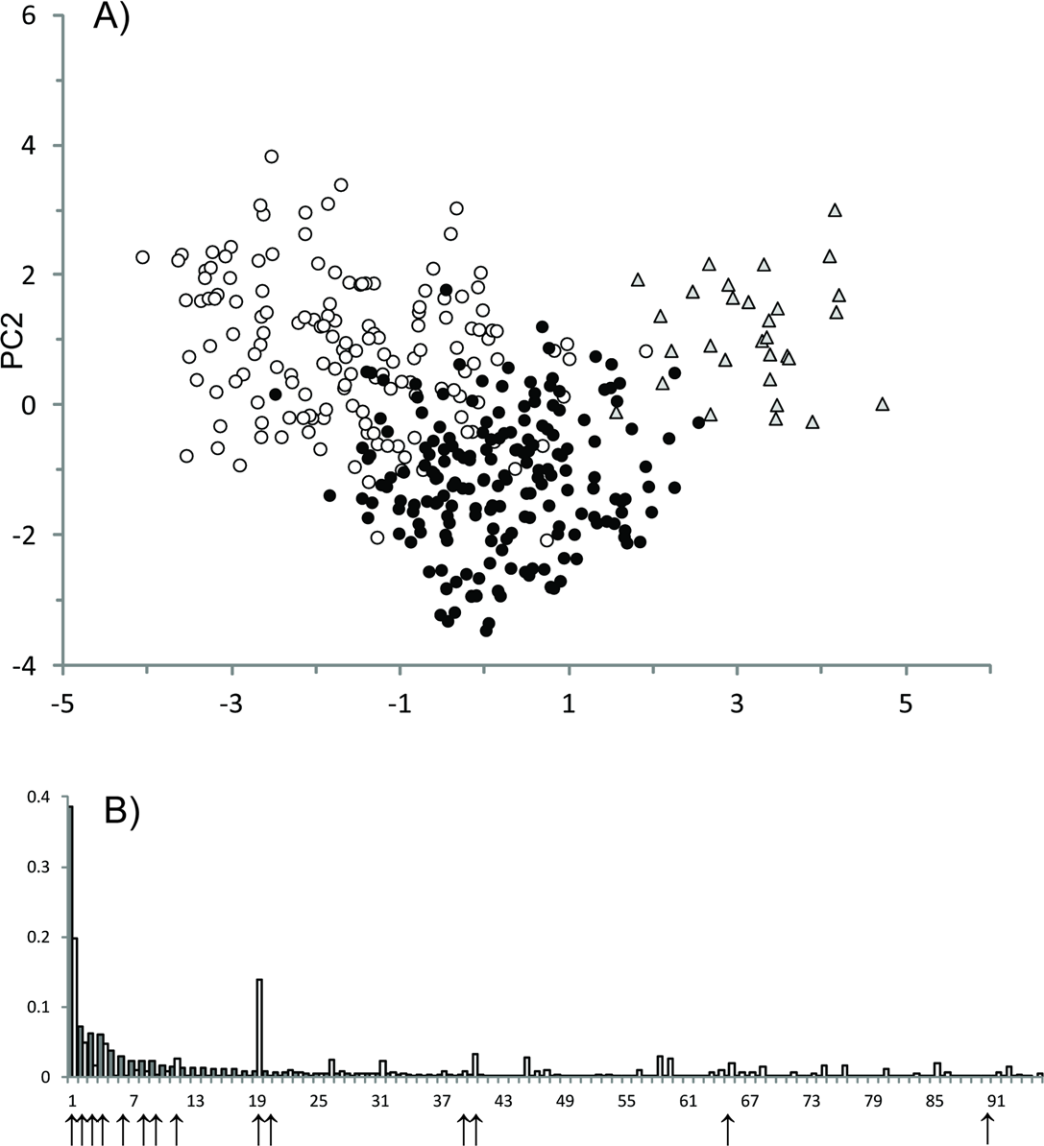
Genotypes form three ecotype associated clusters strongly driven by outlier loci. (A) DAPC clustering for the first two principal components (PCs) explaining 98% of the variation. Filled black dots: Baltic Sea AS; open circles: Baltic Sea SS; grey triangles: North Sea AS. See Table 1 for sample details. (B) proportion of variation contributed to PC1 (grey bars) and PC2 (open bars) by individual loci. Outlier loci are indicated by vertical arrows.

When SNP data alone were analysed for the ten Baltic and North Sea collections, global population divergence was estimated at 0.019, varying between 0 and 0.35 across 95 loci. The DAPC model suggested that SNP variation could be described by three clusters that to a large extent corresponded with three spawning-types 1) Baltic AS, 2) Baltic SS and 3) North Sea AS. Examination of posterior membership probabilities showed that the genetic classification of an individual into the three clusters overall corresponded with its collection location. Thus, across the ten collections, 363 of 400 individuals (91%) were correctly classified to their spawning-type cluster. Inspection of PCs showed that PC1 (explaining 54% variation) differentiated Baltic Sea from North Sea collections, but with closer relationships between North Sea AS and Baltic Sea AS populations than North Sea AS and Baltic Sea SS populations (Fig 3A). PC2 (explaining 44% variation) differentiated Baltic Sea AS from North Sea AS and Baltic Sea SS. Examining how individual SNP loci contributed to PCs showed that 11 loci, of which eight were outlier loci, collectively contributed 75% of the variation in PC1 (Fig 3B). One locus (Cha_15360.2–279) alone explained almost 40% of the total variation. For PC2, the same locus explained 19%. For PC2, the five top ranking loci contributing to clustering (representing in total 47% of the variation) were all outliers.

A *post hoc* PCA using only 15 loci, 14 outlier loci from [14] and the additional outlier detected here, to describe genetic relationships among samples showed that the first axis separated Baltic from North Sea populations, whereas the second axis separated SS and AS populations (S1 Fig). In contrast, using information for 80 neutral loci, samples clustered together with no discernible pattern (S2 Fig), and *post hoc* analyses of neutral sample differentiation only returned five statistically significant pairwise comparisons, three of which involved Bothnian Bay SS versus GoR AS samples and two which involved Northern North Sea AS versus GoR SS (not shown).

Repeating the DAPC analysis where information was initially excluded for 19 non-spawning fish distributed across collections and then ‘assigning’ non-spawners to population clusters, showed that in most cases non-spawning fish clustered closest with the population they were caught with. Thus, 12 of 14 fish collected in autumn assigned to the Baltic Sea AS clusters; one showed closest grouping with North Sea AS, and one showed closest grouping with Baltic Sea SS. All five non-spawning fish collected in spring assigned to Baltic Sea SS (S3 Fig). Therefore the inclusion of non-spawning fish should not have seriously biased estimates of sample differentiation.

## Discussion

We show that herring spawning in spring and autumn in one distinct sub-system of the Baltic Sea, the Gulf of Riga, are genetically highly differentiated. Differentiation was observed for three SNPs and for one transcriptome derived microsatellite marker, but clear population separation was only detectable with SNP markers, and neutral marker frequencies exhibited statistically non-significant differentiation among collections. Thus, the two ecotypes can overall be correctly classified based on 15 SNP markers, of which previously identified outlier loci exhibit strong explanatory power. In contrast to [14] only three SNP loci behaved as statistical outliers in Gulf of Riga samples. The sensitivity of outlier testing is affected by the number of populations and markers in the analysis [39], both of which were considerably lower in our analysis compared to [14]. Thus, the limited number of outlier loci identified here was at least to some extent expected to be caused by increased type II error, and we therefore tentatively classified all loci having exhibited outlier behaviour in Baltic Sea populations either here or in [14] as outliers.

Allele frequency shifts at outlier loci are expected to reflect selective responses caused by strong ecological gradients leading to local adaptation, either for directly associated genes or genes in linkage with the markers (‘genetic hitchhiking’). Although the mechanisms underlying outlier differentiation may vary (reviewed in [3]) and are unknown in the present study, a likely explanation is that outliers reflect a selective response in one or more genes, likely associated with spawning site preference or phenology. The SNP locus explaining most of the variation among clusters (*Cha_15360*.*2–279*) is annotated to a skeletal muscle troponin-coding gene, and another outlier SNP exhibiting strong divergence among clusters (*Cha_1025*.*1–149*) annotates to a ribosomal protein. However, whether these specific gene sequence variations have any causative effects remains to be determined. We hypothesise that Baltic autumn spawning herring may harbour one or more ‘genomic islands’ linked to ecotype divergence (c.f. [42]), in this case potentially associated with adaptation to autumn spawning.

Our results show that Baltic autumn-spawners exhibit the closest genetic relationships with Baltic spring spawning populations. This suggests that all herring sampled in the Gulf of Riga, spring and autumn spawners, from both gulf and open sea, originate from the same post-glacial colonisation event. We did not examine whether samples represented one or more of the three phylogenetic clades represented in all Baltic collections examined to date [21].

Strikingly, for a handful of outlier SNP loci Gulf of Riga autumn-spawners showed closer genetic similarity with autumn spawning populations from the North Sea, which are both geographically distant (>2,000 km shortest waterway) and demographically isolated [43]. Our results could thus point to, that autumn-spawners both in the North Sea and the Baltic Sea are affected by some common selective driver that has led to convergent allele frequencies for specific outlier loci. Convergence in gene frequencies among geographically disjoint populations of divergent ecotypes is observed in other marine fishes, such as threespine stickleback, *Gasterosteus aculeatus* [43] and Atlantic cod, *Gadus morhua* [44, 45]. However, it is often unresolved whether molecular similarities may in fact be the result of shared ancestry rather than of recurrent selective sweeps (e.g. [42]). Lamichhaney et al. [16] found SNP allele frequencies that were similar between North Sea autumn-spawners and a Western Baltic autumn spawning population. However, as Western Baltic herring in general show closer genetic relationships with North Sea populations than with other Baltic Sea populations (e.g. [14,16]), our results thus constitute a separate line of evidence that spawning time divergence may be associated with divergent selection at the genomic level. We have not demonstrated that spawning time differentiation is the main driver of genetic differentiation. Nonetheless, the strong covariance between spawning traits and genotypes shows that although spawning time is a plastic trait in this opportunistic species (e.g. [26]), reproductive isolation may play a role in maintaining sympatric ecotypes.

The adaptive value of adopting a specific spawning time is believed to be related to trade-offs between energy spent on migration and spawning, as well as optimising environmental conditions experienced by developing offspring [46]. In herring, the timing of first spawning is highly plastic and triggered by both body condition and environmental conditions, such as water temperature [47]. Environmental variance may thus affect the optimal timing for spawning both spatially [48] and temporally [49]. Alternative strategies with respect to spawning time are believed to have arisen as opportunistic response to environmental variance that may, or may not, be maintained in separate evolutionary trajectories [50]. The relative strength of sympatric herring ecotypes conversely appears to be linked to complex interplay between feeding conditions, climatic effects and competition acting on fecundity and recruitment. This may lead to alternation in the competitive advantage and fitness of ecotypes ([49], also see [51]). In the Gulf of Riga, the relative predominance of ecotypes is reflected in fisheries data [30]. While spring-spawners mainly contribute to herring landings today the importance of autumn-spawners has decreased dramatically over time. For instance, in the 1950s-1970s autumn-spawners in some years accounted for nearly half the catches [23] but now make up less than 1% of landings (H. Shpilev, unpubl. data). These changes are also reflected in autumn-spawners exhibiting lower individual fecundity [52] and decreased abundance [53], which may to some extent be linked to less favourable environmental (incl. temperature and salinity) conditions for autumn spawners [54]. The perception that Gulf of Riga herring currently thrives is thus driven entirely by proliferation of spring spawning herring, hiding dramatic decreases in the abundance of autumn-spawners. In spite of being comprised of populations with different ecologies, spatio-temporal dynamics [23] and genetic profiles (this study), herring in the Gulf of Riga are exploited and managed as a single stock unit [55]. Our results suggest that in case of complete loss of the autumn spawning herring populations, whether due to climate change, overexploitation, eutrophication or a combination hereof, herring with genotypes enabling the proliferation in autumn will be lost from the system, decreasing the diversity and robustness of the ecosystem [56]. This underpins that failing to separately incorporate all ecologically and evolutionary significant components weakens our understanding of ecosystem processes and may ultimately lead to a suboptimal management of living marine resources.

## Supporting Information

S1 Fig Principal component analysis plot of PC1 and PC2 for 14 outlier SNPs in ten herring collections. (DOCX)

S2 Fig Principal component analysis plot of PC1 and PC2 for 81 neutral SNPs typed in two North Sea and eight Baltic Sea herring collections. (DOCX)

S3 Fig Discriminant analysis of principle components plot of PC1 and PC2 showing genetic relationships for 19 non-spawning herring in a model where only spawning fish were used to build clusters. (PDF)

S1 Table Single Nucleotide Polymorphism and microsatellite loci in the analysis. (DOCX)

S2 Table Genetic marker summary data. (DOCX)
