# Supplementary material for: Outlier Loci Detect Intraspecific Biodiversity amongst Spring and Autumn Spawning Herring across Local Scales
Source: PLoS One. 2016 Apr 6;11(4):e0148499. doi: 10.1371/journal.pone.0148499 (PMC4822851; doi:10.1371/journal.pone.0148499)
Supplement: S1 Fig — (DOCX) [file pone.0148499.s001.docx]

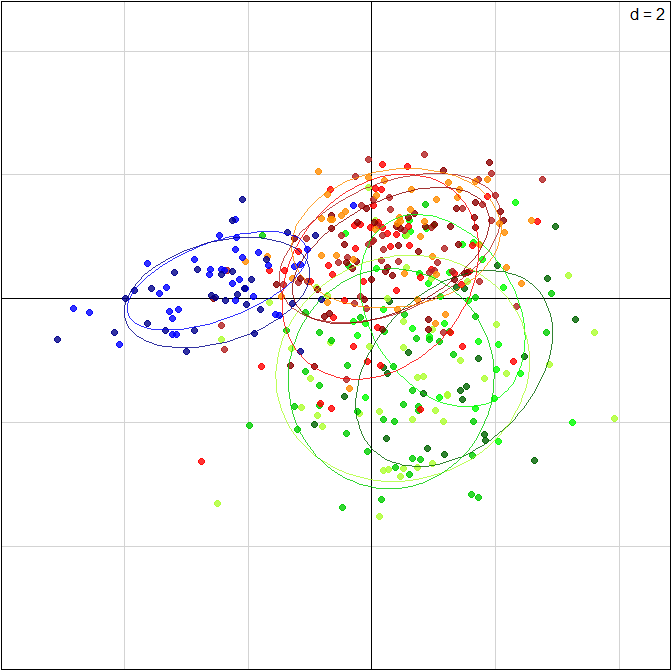


Supporting information Figure 1. PCA for 15 outlier SNPs in ten herring collections. Colour schemes indicate collection origin as follows, blues: North Sea AS; reds: Baltic Sea AS; greens: Baltic Sea SS. Inertia ellipses are given for each collection. Ellipses are centered by the mean PC coordinates and widths represent the variances of the PC coordinates. Individual collections are not identified for better interpretation.
