# Supplementary material for: Outlier Loci Detect Intraspecific Biodiversity amongst Spring and Autumn Spawning Herring across Local Scales
Source: PLoS One. 2016 Apr 6;11(4):e0148499. doi: 10.1371/journal.pone.0148499 (PMC4822851; doi:10.1371/journal.pone.0148499)
Supplement: S2 Fig — (DOCX) [file pone.0148499.s002.docx]

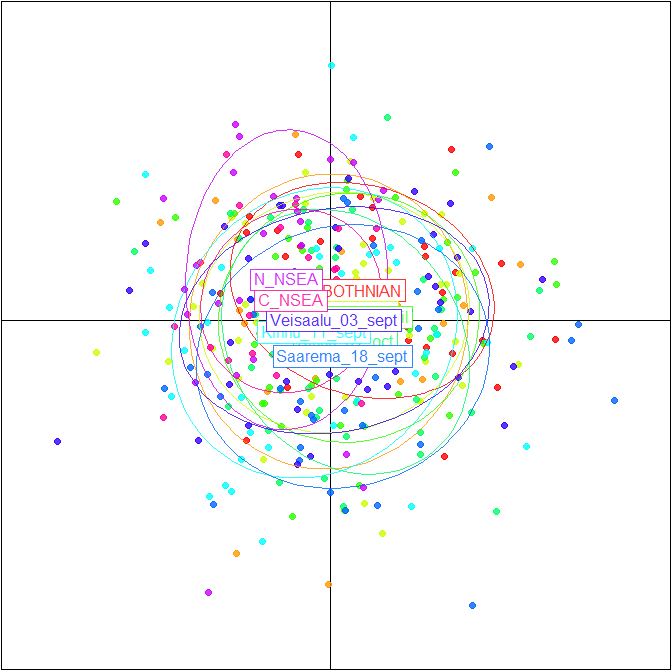


Supporting information Figure 2. PCA for 80 neutral SNPs typed in two North Sea and eight Baltic Sea samples, each shown with a different colour code. Collection name, as identified in Table 1, are indicated together with inertia ellipses for each collection. Ellipses are centered by the mean PC coordinates and widths represent the variances of the PC coordinates.
