# Supplementary figures and images for: Outlier Loci Detect Intraspecific Biodiversity amongst Spring and Autumn Spawning Herring across Local Scales
Source: PLoS One. 2016 Apr 6;11(4):e0148499. doi: 10.1371/journal.pone.0148499 (PMC4822851; doi:10.1371/journal.pone.0148499)

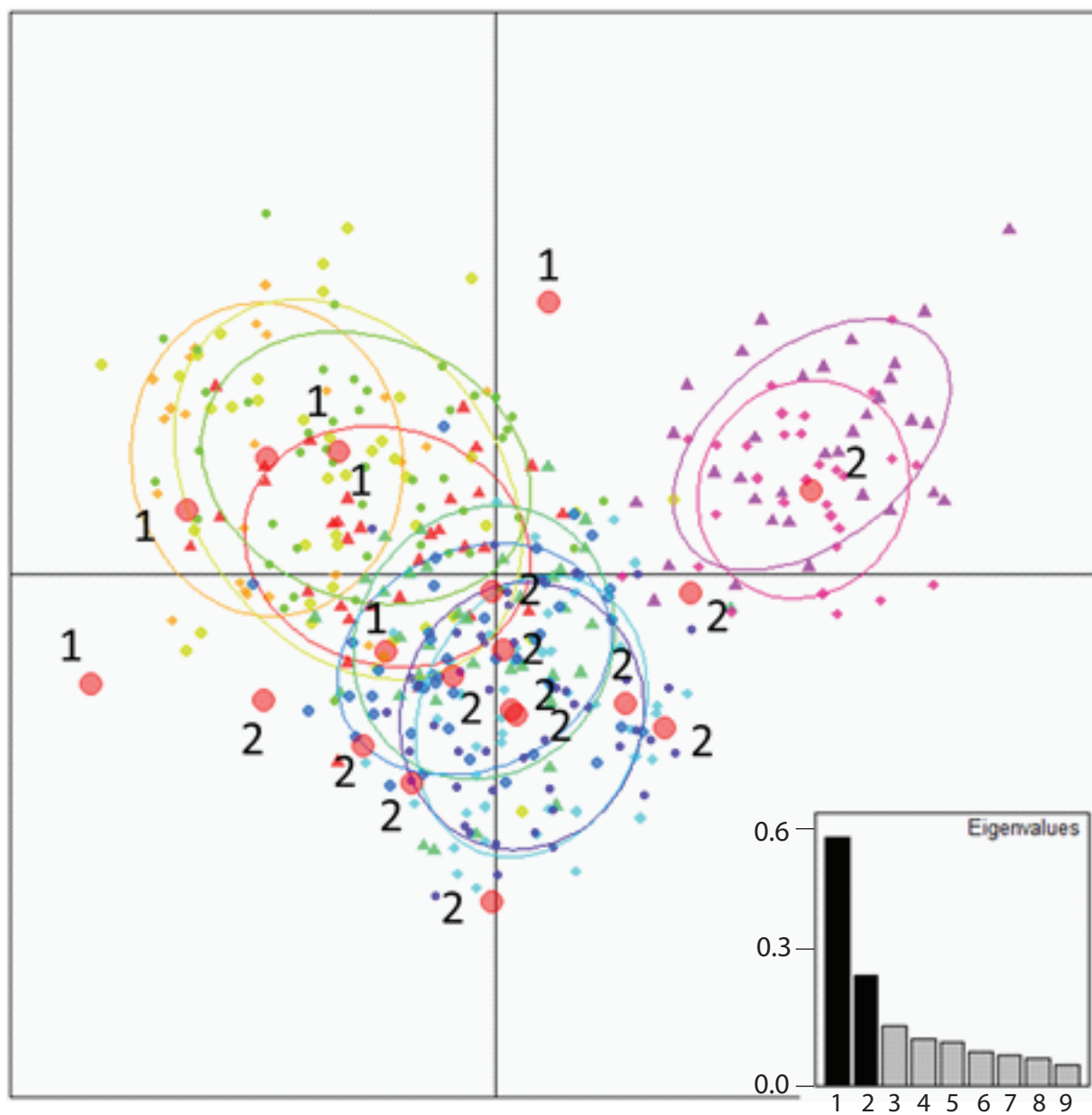

Supplement: S3 Fig — (PDF) [file pone.0148499.s003.pdf]
