# Supplementary material for: Outlier Loci Detect Intraspecific Biodiversity amongst Spring and Autumn Spawning Herring across Local Scales
Source: PLoS One. 2016 Apr 6;11(4):e0148499. doi: 10.1371/journal.pone.0148499 (PMC4822851; doi:10.1371/journal.pone.0148499)
Supplement: S1 Table — (DOCX) [file pone.0148499.s004.docx]

Supporting Table 1. Loci genotyped in the analysis, ranked highest to lowest by estimated differentiation across six GoR samples. SNP loci are from Helyar et al. (2012) and microsatellite loci are from Teacher et al. (2011). SNP loci identified by Limborg et al. (2012) as selective outliers across Baltic Sea and North Sea populations are indicated by #. Three loci identified as outliers in the present study are indicated by *

| marker type | **Locus** | **Rank global theta** |
| --- | --- | --- |
| SNP | Cha_15360.2_279# * | 1 |
| SNP | Cha_7833.1_97 * | 2 |
| SNP | Cha_381.2_437# * | 3 |
| SNP | Cha_15898.2_568 | 4 |
| SNP | Cha_16335.1_144 | 5 |
| SNP | Cha_16330.7_357# | 6 |
| SNP | Cha_2884.1_367# | 7 |
| SNP | Cha_297.1_93# | 8 |
| SNP | Cha_10193.1_449# | 9 |
| SNP | Cha_7143.1_573 | 10 |
| SNP | Cha_15984.1_275# | 11 |
| SNP | Cha_15659.7_503 | 12 |
| SNP | Cha_1143.1_484 | 13 |
| SNP | Cha_16319.1_575 | 14 |
| SNP | Cha_874.1_105 | 15 |
| SNP | Cha_7438.1_87 | 16 |
| SNP | Cha_159.1_649 | 17 |
| SNP | Cha_800.1_127 | 18 |
| SNP | Cha_2168.1_289 | 19 |
| SNP | Cha_1473.1_437 | 20 |
| SNP | Cha_5534.1_506# | 21 |
| SNP | Cha_1068.2_349# | 22 |
| SNP | Cha_2115.3_303 | 23 |
| microsatellite | Her25 | 24 |
| microsatellite | Her142 | 25 |
| SNP | Cha_4850.1_589 | 26 |
| SNP | Cha_4723.1_99 | 27 |
| SNP | Cha_14920.3_464 | 28 |
| SNP | Cha_143.1_185# | 29 |
| SNP | Cha_1400.3_301 | 30 |
| SNP | Cha_7205.2_138 | 31 |
| SNP | Cha_15117.2_376 | 32 |
| SNP | Cha_590.3_373 | 33 |
| SNP | Cha_8800.1_246 | 34 |
| SNP | Cha_279.9_165 | 35 |
| SNP | Cha_140.3_229 | 36 |
| SNP | Cha_14751.1_172 | 37 |
| SNP | Cha_12617.1_224 | 38 |
| SNP | Cha_8506.1_89 | 39 |
| SNP | Cha_12381.1_143 | 40 |
| SNP | Cha_12119.7_664 | 41 |
| microsatellite | Her59 | 42 |
| microsatellite | Her140 | 43 |
| SNP | Cha_14490.1_120 | 44 |
| microsatellite | Her141 | 45 |
| SNP | Cha_372.1_168 | 46 |
| microsatellite | Her114 | 47 |
| microsatellite | Her71 | 48 |
| SNP | Cha_160.1_805 | 49 |
| microsatellite | Her67 | 50 |
| microsatellite | Her77 | 51 |
| SNP | Cha_221.1_314 | 52 |
| SNP | Cha_9821.1_89 | 53 |
| SNP | Cha_11896.1_201 | 54 |
| microsatellite | Her21 | 55 |
| SNP | Cha_12154.1_272 | 56 |
| SNP | Cha_926.1_253 | 57 |
| SNP | Cha_11341.1_150 | 58 |
| SNP | Cha_10564.1_104 | 59 |
| SNP | Cha_4453.1_213 | 60 |
| SNP | Cha_14942.1_236 | 61 |
| SNP | Cha_10060.1_106 | 62 |
| SNP | Cha_3032.1_626 | 63 |
| SNP | Cha_10864.1_91 | 64 |
| SNP | Cha_15995.1_148 | 65 |
| SNP | Cha_11197.1_111 | 66 |
| SNP | Cha_7075.1_58 | 67 |
| SNP | Cha_462.3_102 | 68 |
| microsatellite | Her143 | 69 |
| microsatellite | Her133 | 70 |
| microsatellite | Her126 | 71 |
| SNP | Cha_6327.1_290 | 72 |
| SNP | Cha_15936.1_224 | 73 |
| microsatellite | Her12 | 74 |
| SNP | Cha_11791.1_247 | 75 |
| SNP | Cha_205.3_223 | 76 |
| SNP | Cha_15656.1_489 | 77 |
| SNP | Cha_11434.1_589 | 78 |
| SNP | Cha_11018.1_175 | 79 |
| SNP | Cha_3028.2_192 | 80 |
| SNP | Cha_8757.1_443 | 81 |
| SNP | Cha_659.1_1361 | 82 |
| SNP | Cha_41.1_1026 | 83 |
| SNP | Cha_13097.1_122 | 84 |
| SNP | Cha_14060.1_125 | 85 |
| SNP | Cha_1030.1_438 | 86 |
| SNP | Cha_1981.1_199 | 87 |
| microsatellite | Her36 | 88 |
| SNP | Cha_16057.1_75 | 89 |
| SNP | Cha_957.1_409 | 90 |
| microsatellite | Her100 | 91 |
| SNP | Cha_152.1_99 | 92 |
| SNP | Cha_209.4_258 | 93 |
| SNP | Cha_12771.1_298 | 94 |
| SNP | Cha_13259.1_167# | 95 |
| SNP | Cha_52.1_173 | 96 |
| SNP | Cha_11309.1_174 | 97 |
| SNP | Cha_1203.1_312 | 98 |
| SNP | Cha_2573.1_161 | 99 |
| SNP | Cha_9634.1_256 | 100 |
| SNP | Cha_124.5_901 | 101 |
| microsatellite | Her117 | 102 |
| SNP | Cha_2.1_256 | 103 |
| microsatellite | Her1 | 104 |
| SNP | Cha_1165.2_123# | 105 |
| SNP | Cha_11286.1_153 | 106 |
| SNP | Cha_15105.2_341# | 107 |
| SNP | Cha_13178.2_124# | 108 |
| SNP | Cha_13439.1_183 | 109 |
| SNP | Cha_224.4_447 | 110 |
| SNP | Cha_3032.3_221 | 111 |
| SNP | Cha_9008.1_90 | 112 |
| SNP | Cha_11053.1_152 | 113 |
| SNP | Cha_8386.6_423 | Failed |
| microsatellite | Her114 | Failed |
