# Supplementary material for: Outlier Loci Detect Intraspecific Biodiversity amongst Spring and Autumn Spawning Herring across Local Scales
Source: PLoS One. 2016 Apr 6;11(4):e0148499. doi: 10.1371/journal.pone.0148499 (PMC4822851; doi:10.1371/journal.pone.0148499)
Supplement: S2 Table — (DOCX) [file pone.0148499.s005.docx]

Supporting information Table 2. Genetic marker summary data for *Clupea harengus* collections (each 48 fish) from the Gulf of Riga. Numbers give averages across respectively 95 SNP and 18 microsatellite loci.

| Collection | Allele count | Allelic richness | H_expected_ | H_observed_ |
| --- | --- | --- | --- | --- |
| Pärnu Bay 24.04.2014 (SS) | 2/7.5 | 2.00/5.68 | 0.410/0.579 | 0.388/0.523 |
| Pärnu Bay 30.04.2014 (SS) | 2/6.9 | 2.00/5.45 | 0.407/0.556 | 0.401/0.518 |
| Kihnu 11.09.2014 (AS) | 2/7.0 | 2.00/5.55 | 0.405/0.568 | 0.391/0.544 |
| Kihnu 01.10.2014 (AS) | 2/7.4 | 2.00/5.90 | 0.403/0.571 | 0.395/0.529 |
| Saarema 03.09.2014 (AS) | 2/6.8 | 2.00/5.35 | 0.405/0.560 | 0.395/0.543 |
| Saarema 18.09.2014 (AS) | 2/6.9 | 2.00/5.62 | 0.405/0.565 | 0.388/0.533 |
